# The Efficacy of Graphene Foams for Culturing Mesenchymal Stem Cells and Their Differentiation into Dopaminergic Neurons

**DOI:** 10.1155/2018/3410168

**Published:** 2018-06-03

**Authors:** Nishat Tasnim, Vikram Thakur, Munmun Chattopadhyay, Binata Joddar

**Affiliations:** ^1^Inspired Materials & Stem-Cell Based Tissue Engineering Laboratory (IMSTEL), Department of Metallurgical, Materials and Biomedical Engineering, University of Texas at El Paso, 500 W University Avenue, El Paso, TX 79968, USA; ^2^Department of Biomedical Sciences, Center of Emphasis in Diabetes and Metabolism, Texas Tech University Health Sciences Center, 5001 El Paso Drive, El Paso, TX 79905, USA; ^3^Border Biomedical Research Center, University of Texas at El Paso, 500 W University Avenue, El Paso, TX 79968, USA

## Abstract

The implantation of stem cells in vivo is the ideal approach for the restoration of normal life functions, such as replenishing the decreasing levels of affected dopaminergic (DA) neurons during neurodegenerative disease conditions. However, combining stem cells with biomaterial scaffolds provides a promising strategy for engineering tissues or cellular delivery for directed stem cell differentiation as a means of replacing diseased/damaged tissues. In this study, mouse mesenchymal stem cells (MSCs) were differentiated into DA neurons using sonic hedgehog, fibroblast growth factor, basic fibroblast growth factor, and brain-derived neurotrophic factor, while they were cultured within collagen-coated 3D graphene foams (GF). The differentiation into DA neurons within the collagen-coated GF and controls (collagen gels, plastic) was confirmed using *β*-III tubulin, tyrosine hydroxylase (TH), and NeuN positive immunostaining. Enhanced expression of *β*-III tubulin, TH, and NeuN and an increase in the average neurite extension length were observed when cells were differentiated within collagen-coated GF in comparison with collagen gels. Furthermore, these graphene-based scaffolds were not cytotoxic as MSC seemed to retain viability and proliferated substantially during in vitro culture. In summary, these results suggest the utility of 3D graphene foams towards the differentiation of DA neurons from MSC, which is an important step for neural tissue engineering applications.

## 1. Introduction

Among all organs in our body, the human brain is one of the largest and most complex, consisting of 100 billion nerves that communicate via trillions of synaptic connections [1]. In the past, the brain was thought to be a slowly decaying organ [1], which can form the basis of several neurodegenerative disorders [1]. Several studies now suggest that stem cells can be isolated and used to restore function in the adult brain, such as dopamine-producing dopaminergic (DA) neurons that are formed in the adult substantia nigra [1]. Implantation of such patient-specific stem cell-derived DA neurons and their regenerative responses might provide a path to functional recovery in neurodegenerative disease and brain injury [2]. The value of a lab-created DA neuronal tissue in a dish using a patient's own stem cells is immense, including in vitro modeling and regenerative medicine [3]. First, tissues created from the patient's own mesenchymal stem cells can be potentially transplanted back without ethical or immunological challenges [3, 4]. Second, such tissue on dish models can serve as better platforms for clinical drug testing generally performed in animals, which sometimes have drastically different outcomes compared to clinical trials [4]. In addition, a lab-created tissue on a dish that accurately mimics actual brain tissue would be significant for researching not only the effect of drugs but neurodegenerative disorders like Parkinson's, Alzheimer's, and ALS (amyotrophic lateral sclerosis) as well [5–11]. So, our long-term goal is to culture 3D DA neuronal tissues on a dish that can capture in vivo neuronal functions and can be useful as tissue-on-a-chip for drug cytotoxicity studies. At the same time, we are interested in providing an optimal 3D scaffold for evaluating the adhesion, culture, and differentiation of mesenchymal stem cells into DA neurons, for use as a platform for neural tissue engineering applications, such as for the treatment for neurodegenerative disorders.

Biomimetic 3D scaffolds are preferred tools for culturing neurons as they provide defined mechanical and physicochemical properties with an interconnected porous structure that can enable a higher or more complex organization than traditional two-dimensional monolayer conditions [12]. Changes in the internal geometry and mechanical properties of such 3D scaffolds can impact cell behavior including survival, growth, and cell fate choice [12]. Other specific characteristics required of scaffolds for culturing neurons are electroconductivity and nanoarchitecture, both of which are offered by graphene [13, 14]. Graphene is composed of a single layer of carbon atoms arranged in a two-dimensional honeycomb lattice [15]. Other than being routinely used for electrical, optical, and thermal applications, studies also proposed the potential of graphene for biomedical applications [15]. Graphene can be used as an optimized scaffold for cell culture, tissue engineering, and regenerative medicine applications [13]. Published works of others have shown that graphene substrates can support the adhesion, proliferation, and differentiation of mesenchymal stem cells (MSCs), induced pluripotent stem cells (iPSC), and other mammalian cells [13]. Specifically for neural tissue regeneration, graphene has demonstrated the ability to perform as an effective platform compatible with neural cells or their precursors [13] and promoted neurogenesis, as assessed by neurite sprouting and neural network formation [14, 16]. Human MSC growth, followed by neural differentiation, was also supported by a monolayer of graphene substrate [17]. Further, the capability of graphene substrates to electrically stimulate differentiated neuronal cells was demonstrated [18]. Our recent published work showed that graphene-oxide coatings enhanced the survival and proliferation of SH5YSY neuronal cells [19]. Based on these reports, we hypothesized that graphene-based substrates may be a promising scaffold material for neural tissue engineering.

In this study, our objective was to utilize a commercially available 3D graphene scaffold termed as “graphene foam” (GF) for culturing mouse MSCs and differentiating them into DA neurons. We hypothesized that these MSC-differentiated DA neurons when cultured in a 3D scaffold will more closely exhibit morphologies, functions, and other necessary characteristics of in vivo DA neuronal tissues, compared to 2D culture or monolayer substrates. To culture cells on the hydrophobic graphene substrates [20], they need to be coated with proteins, such as laminin [21, 22], to promote hydrophilicity and cell adhesion onto these surfaces. On the other hand, collagen coating is a well-established procedure for cell culture, and collagen coatings when applied to graphene-based substrates were shown to not interfere with the porous structure of graphene [23]. So, we opted to coat the hydrophobic graphene foams using collagen as it would lead to the formation of a hydrophilic, porous, and conductive scaffold ideal for neuron culture.

This work will significantly contribute by enabling a platform that will allow us to study interactions between healthy DA neurons and their synaptic communications and identify mechanisms involved in DA neuron apoptosis during injury and disease. As studying DA neuronal cell death in human brains is extremely difficult and invasive, the development of such in vitro 3D models of DA neurons would make it feasible to probe cellular and molecular mechanisms of neurodegenerative disorders and implement novel therapeutic strategies. This study is innovative as the technique might be adaptable for engineering other 3D tissue models from different stem cell types.

## 2. Materials and Methods

### 2.1. Preparation of the Graphene Foam and Collagen Coating

3D multilayer freestanding graphene foams (GF) (2^″^ × 2^″^) were purchased from Graphene Supermarket (Calverton, NY). For cleaning, these foams were washed with 70% ethanol followed by UV exposure for 30 min in a laminar sterile flow hood. Using a sterile biopsy punch (~1 mm deep, 8 mm in diameter), samples were prepared for further processing and experimentation. These pristine GF discs were collagen coated [24] and cross-linked with genipin [25], using published guidelines. For coating of the GF, collagen from bovine achilles tendon (Sigma-Aldrich) at a concentration of 9 mg/ml in 0.2 M acetic acid was used for extraction of acid-soluble collagen (for 24 hr at 200 rpm). The extract was analyzed using Fourier transform infrared spectroscopy (FTIR) to confirm the presence of collagen, in comparison with existing literature.

After collagen coating, GF samples were incubated at 37°C for 24 hr in genipin (stock solution of 100 mM in DMSO, Enzo Life Sciences), prepared using a ratio of 1 : 100 of genipin in 1x PBS to further cross-link the collagen atop the GF [26]. After 24 hr, the cross-linked collagen-coated GF samples were washed using sterile 1x PBS (Sigma-Aldrich) for 3 times prior to consecutive experiments.

To confirm the cross-linking of the collagen using genipin atop the GF using the procedure described above [26], rheometry was used to compare the properties of the non-cross-linked versus the cross-linked collagen samples. Collagen gels for rheometry were formed as described previously [27] and cut using a biopsy punch (~1 mm deep, 8 mm in diameter). The gels were preswollen in 1x PBS before testing. Oscillatory shear stress rheometry was performed (1% strain, 0.5–50 Hz) using an Anton-Paar MCR101 rheometer (Anton-Paar, Graz, Austria) with an 8 mm parallel plate geometry. The strain and frequency range were analyzed within the linear viscoelastic range of the gels by frequency sweeps. Elastic modulus was calculated through complex shear modulus with storage and loss modulus, and complex viscosity was measured at 1.76–1.99 Hz for all samples, as done earlier [27].

### 2.2. Material Characterization

#### 2.2.1. Scanning Electron Microscopy (SEM)

Images of the surfaces of the pristine GF were acquired using SEM (S-4800, Hitachi, Japan) at voltages of 10 kV. For imaging of the collagen-coated GF, samples were air-dried and coated with graphite spray (Electron Microscopy Sciences, Hatfield, PA) to minimize charging during observation and imaged at voltages of 1 kV.

#### 2.2.2. Raman Analysis

The pristine GF and collagen-coated GF were characterized by Raman spectroscopy to study the vibrational properties of the material to provide information on molecular vibrations and crystal structures.

#### 2.2.3. X-Ray Diffraction Analysis

For the phase analysis, the samples were air-dried prior to X-ray diffraction (XRD, D8 DISCOVER, Bruker's diffractometer, Karlsruhe, Germany). XRD was carried out at 40 kV voltage and 40 mA current with CuK*α* wavelength (1.54056 Å) and 2*θ* ranging from 10° to 50° at a scanning rate of 3°/min with a step size of 0.1°.

#### 2.2.4. Electrical Characterization

To explore the electrical transport properties of GF and collagen-coated GF, a two-probe measurement was conducted using a micromanipulator (Carson City, Nevada). In the measurements, tungsten probes were used to measure the *I*-*V* (current versus voltage) curve when a bias voltage of 0 to 3 V was applied.

### 2.3. Biocompatibility of the Collagen-Coated Graphene Foams

Strain C57BL/6 Mouse Mesenchymal Stem Cells (mouse MSC, catalog number: MUBMX-01001) and Mouse Mesenchymal Stem Cell Growth Medium (complete growth medium, catalog number: MUXMX-90011) were purchased from Cyagen (Santa Clara, CA, USA). The cells were grown and stabilized for at least 8 passages before being used in further experiments. Prior to being introduced into the 3D scaffolds, cells were labeled with PKH26 red fluorescent dye (Sigma) following the manufacturer's protocols. These labeled mouse MSCs were seeded atop collagen-coated GF or controls (tissue culture plastic wells) in a density of 1 × 10^6^ cells/ml placed within 24 wells of a tissue culture well plate (Thermo Fisher Scientific) and cultured for at least 72 hr (37°C, 5% CO_2_). Confirmation of cell retention within the collagen-coated GF was done using SEM (as described before) and inverted confocal fluorescence microscopy (Zeiss LSM 700 Confocal, Germany). To account for absolute cell numbers that remained viable and proliferated within the scaffolds compared with control wells (tissue culture plastic), 3D scaffolds of collagen-coated GF were seeded with 10^3^ mouse MSCs per well in a 96-well plate. To estimate cell proliferation after 48 hours, both gels and wells with cells were gently rinsed with PBS, overlaid with 200 *μ*l of 0.25% trypsin-EDTA per well, and incubated at 37°C for 10 min on an orbital shaker (30 rpm). Extracted cells were pelleted by centrifugation and counted using a hemocytometer.

### 2.4. Flow Cytometry (FACS) Analysis

To estimate cell proliferation and overall biocompatibility of the collagen-coated GF, mouse MSCs were prestained using the CellTrace Violet Cell Proliferation Kit (Invitrogen, Carlsbad, CA, USA) using the manufacturer's protocols. These prestained cells were seeded (4 × 10^6^ cells/ml) atop 3D collagen-coated GF and 2D tissue culture plastic wells (controls) and cultured for 24 hr and 48 hr, respectively (37°C, 5% CO_2_). After 24 and 48 hr, samples were treated using Trypsin-EDTA (0.25%, phenol red) (Thermo Fisher Scientific, Waltham, MA); then, cells were detached, extracted, and processed for flow cytometry (FACS). Extracted cells were fixed and processed further for FACS (Beckman Coulter Gallios Flow Cytometer, Brea, CA, USA) using excitation and emission wavelengths of 405 and 450 nm, respectively. Prestained cells grown in plastic Petri dishes for 72 hr served as positive controls. Negative controls included nonstained cells cultured on plastic Petri dishes for 72 hr.

### 2.5. Differentiation of Mouse MSCs into DA Neurons

Mouse MSCs used for the differentiation were cultured and passaged as described below. Prior to cell seeding, T-75 culture flasks were coated with 0.1% gelatin (Sigma-Aldrich, St. Louis, MO, USA) and incubated (37°C for 1 hr). After this, the cell suspension in complete culture medium was transferred to a gelatin-coated T-75 flask and incubated for 1 hr (37°C, 5% CO_2_, and 95% RH). Prior to cell culture, the gelatin solution used for coating of the flasks was aspirated. After 70% confluency in culture was attained, cells were trypsinized and passaged for further experiments.

For induction of differentiation of mouse MSCs into DA neurons, 2 types of 3D scaffolds were used. These included the collagen-coated GF and collagen gels only. For the preparation of collagen gels, the collagen extract was loaded into a 10 ml syringe (BD Biosciences, San Jose, CA) and ejected into a 24-well plate for deposition and settling. Once a smooth surface of the deposited collagen was observed, the wells were incubated with the genipin solution for cross-linking (as described earlier). After cross-linking was completed, uniform-size disc-shaped samples for both collagen-coated GF and collagen gels only were punched-out using a biopsy punch (~1 mm deep, 8 mm in diameter).

For the differentiation of mouse MSCs into DA neurons atop 3D scaffolds or 2D culture plastic, published protocols were followed [28]. For the differentiation, initial cell seeding density of mouse MSCs in the 2D plastic wells was maintained at 3 × 10^5^ cells/ml based on published guidelines; for the 3D scaffolds, cell density was adjusted based on the total volume of the scaffolds (6 × 10^6^ cells/ml). Briefly, passaged mouse MSCs were seeded on poly-D-lysine- (BD Biosciences) coated dishes (using complete growth media for mouse MSCs), and after 24 hr, the culture medium was replaced using Neurobasal Media (catalog number: 21103049; Thermo Fisher Scientific). At this point, sonic hedgehog (SHH, R&D Systems, Minneapolis, MI), fibroblast growth factor (FGF8, R&D Systems), and basic fibroblast growth factor (BFGF, R&D Systems) were added and incubated for 6 days. After this, brain-derived neurotrophic factor (BDNF, Cell Sciences, Canton, MA) was added to the culture and further incubated for 3 days. After a total of 9 days of culture, to confirm the differentiation of MSCs into DA neurons, the cell-seeded scaffolds and controls were fixed with 4% paraformaldehyde (Sigma) for 15 min (25°C) and then permeabilized with 0.2% Triton X-100/PBS for 1 hr. After blocking with 1% normal goat serum (NGS/PBS, Sigma) for 1 hr at room temperature, the samples were incubated with a mouse monoclonal antibody to *β*-III tubulin [5.2F] to locate *β*-III tubulin and to detect vimentin; the samples were then incubated with a vimentin mouse monoclonal antibody (24 hr at 4°C) followed by a goat polyclonal secondary antibody to mouse IgG1-heavy chain (FITC) (Abcam, Cambridge, UK) (2 hr at 25°C). For the detection of TH, the samples were incubated with a purified rabbit monoclonal IgG antibody to TH; for the detection of NeuN, the samples were incubated with a NeuN rabbit polyclonal antibody (24 hr at 4°C) followed by a goat anti-rabbit IgG secondary antibody, Alexa Fluor 488 conjugate (2 hr at 25°C), at a dilution of 1 : 1000 in the dark. The samples were then washed with 1x PBS thrice and mounted using Fluoromount-G with DAPI (Thermo Fisher Scientific) and imaged using confocal fluorescence microscopy (Olympus IX81 inverted fluorescence motorized microscope, Japan).

As a control, for the differentiation of mouse MSCs into DA neurons, we chose to induce differentiation in human MSCs alongside (online supplement) the mouse MSCs. This was important to confirm the validity of the differentiation induction protocol in mouse MSCs, compared to the human MSCs, and also contrast the response of both cell types to the same differentiation protocol. Further, both mouse and human MSCs were validated by alkaline phosphatase (ALP) staining prior to their differentiation induction, to confirm their stemness and pluripotency (online supplement) [29].

Axonal extensions on proximal and distal sides of differentiated neurons in collagen-coated GF and in collagen were measured by calculating the axonal outgrowth length, visualized with *β*-III tubulin or NeuN and analyzed using ImageJ software [30]. The final results were expressed as the average length of neurite extensions in collagen-coated GF and in collagen, normalized with controls (cells differentiated in 2D wells).

### 2.6. Statistical Analysis

All samples were present in triplicate unless otherwise mentioned. Numerical data are expressed as the mean ± standard deviation. Microsoft Excel Student's *t*-test was performed to determine if the averages of any two sample datasets compared were significantly different. *p* values less than 0.05 were considered significant.

## 3. Results and Discussion

**Figure 1:**
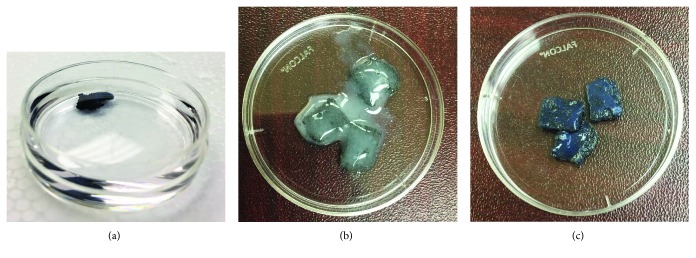
(a) Pristine graphene foam floating in PBS in a 60 × 15 mm Petri dish. (b) Graphene foam being coated with collagen. (c) Graphene foam after the collagen coating was cross-linked with genipin (100 × 15 mm Petri dish shown in (b) and (c)).

As shown in Figure 1(a), the pristine GF was extremely light, hydrophobic, and fragile during routine handling. For this reason, the pristine foams had to be coated with collagen to retain hydrophilicity, increase their weight, and improve their handling characteristics (Figures 1(b) and 1(c)).

**Figure 2:**
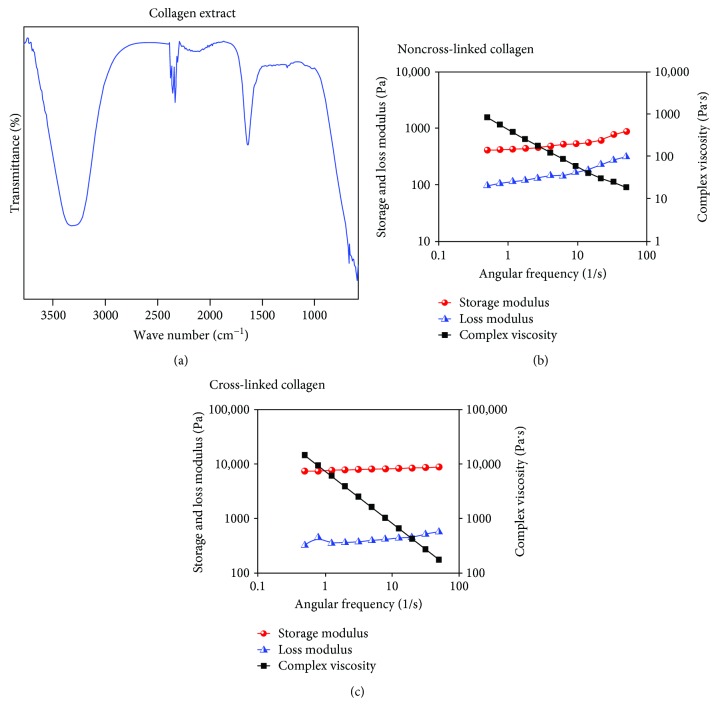
(a) FTIR spectra of the collagen extract. Shown in (b) and (c) are rheological analyses of the non-cross-linked and cross-linked collagen, respectively. Characteristic datasets were obtained from disc-shaped (8 mm) samples of collagen, in both cases.

The FTIR spectra of the acid-soluble collagen extract are shown in Figure 2(a). The hydrogen bonding of the N-H group of the peptide was evident at 3300 cm^−1^ [31]. The amide-I band was evident around 1635 cm^−1^, fitting well the range of 1625–1690 cm^−1^ for the general amide-I band position. This was due to the existence of hydrogen bonds in collagen [31]. The helical structure of the collagen was confirmed from the IR absorption ratio between 1263 (amide-III), which was approximately equal to each preparation. The results showed that the helical structure of collagens was kept well.

It was essential to confirm the cross-linking of the collagen atop the GF by a secondary technique, other than by visual confirmation (Figure 1(c)). Therefore, rheometric analysis of the non-cross-linked and cross-linked collagen samples was done, from which it was determined that the strain and frequency range were within the linear viscoelastic range of the gels by amplitude and frequency sweeps (Figures 2(b) and 2(c)). We were able to generate cross-linked gels of a significantly enhanced elastic modulus of ~5.0 kPa compared to the non-cross-linked samples which revealed an elastic modulus of ~1.78 kPa. Additionally, cross-linking increased the complex viscosity of the gels from 277 to 2610 Pa·s.

**Figure 3:**
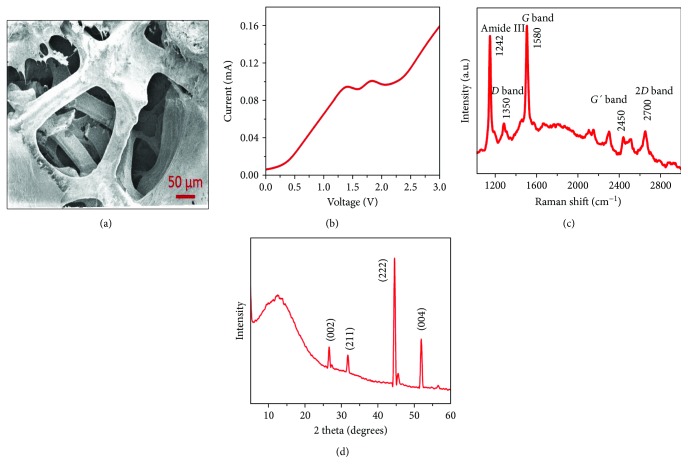
Material characterization of the graphene foam coated with collagen coating and cross-linked with genipin. (a) Scanning electron microscopy (SEM). (b) Current-voltage (*I*-*V*) characteristics. (c) Raman and (d) XRD spectra, respectively.

Figure 3(a) shows a characteristic SEM image of a collagen-coated GF that confirmed the deposited collagen coating, in comparison with the pristine GF (Supplementary Figure 1A). Further, it was evident that the collagen coating did not alter the basic morphology and architecture of the GF.

From the *I*-*V* (current versus voltage) curve, it is clear that the GF exhibits a nonlinear behavior with a current level of ~0.10 A at 3 V (Supplementary Figure 1B), while the collagen-coated GF also exhibited a nonlinear behavior but with three orders of magnitude drop in current (~0.16 mA at 3 V) in Figure 3(b). For the collagen-coated GF, the curve did not appear as smooth as the GF, as collagen is an insulator by nature which introduces noise to the signal. From this measurement, it is clear that the collagen-coated GF yields reasonable electrical transport compared to pristine GF which paves the way for further electric stimulation of neuronal cells.

Figure 3(c) demonstrates the typical Raman spectra of a collagen-coated GF using a 532 nm laser at room temperature. The Raman spectra of the 3D GF contained two major peaks near 1580 and 2700 cm^−1^, corresponding to the *G* and *2D* bands of graphene (Supplementary Figure 1C). There is no major *D* band in the Raman spectra of the pristine GF, which confirmed that it is almost defect-free. The integrated intensity ratio of the *G* to *2D* band (*G*/*2D*) indicates that the GF was primarily multilayered graphene [32]. After coating the GF with collagen, *D* peak appeared near 1350 cm^−1^ and some other peaks emerged including a minor peak at ~2450 cm^−1^ (*G*′ band). The defect was increased from 0.02 to 0.7 in collagen-coated GF which is estimated from the intensity ratio of the *D* band and *G* band (*D*/*G*) [33]. The amide-III peak appeared at 1242 cm^−1^ which is a characteristic of the collagen (Figure 3(c)), though the amide-I and amide-II peaks were absent in the spectra [34]. These results confirmed the successful deposition of collagen atop 3D GF.

XRD spectra of the collagen-coated GF is shown in Figure 3(d) and was indexed as described. The two diffraction peaks at 2*θ* = 26.5° and 2*θ* = 55° correspond to the (002) and (004) planes of graphene, respectively, where the intensity of the peak at 2*θ* = 26.5° got reduced for the collagen coating [33, 35]. The diffraction peaks of collagen appeared to correspond to the crystallographic planes (211) and (222) at about 2*θ* = 32° and 2*θ* = 45.3° indicating a traditional mineralized collagen [36]. The X-ray diffraction (XRD) peaks of collagen-coated GF confirmed that they existed in separate planes. In addition, they did not interact with each other which is supported by their distinct peak positions without any considerable change that is supported by the above references [33, 35, 36].

**Figure 4:**
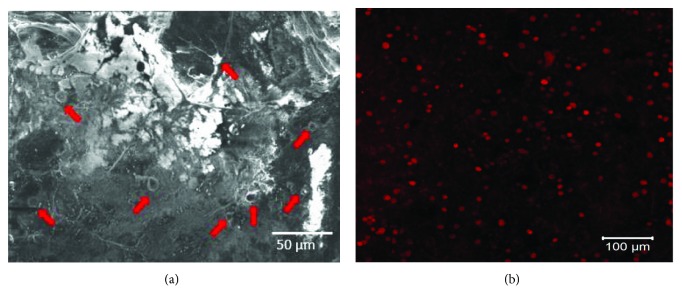
Adhesion and retention of mesenchymal stem cells cultured in collagen-coated GF shown by (a) SEM imaging and (b) fluorescent images of PKH26-prestained cells within the scaffold. Red arrows in (a) point to the cells and their extension processes.

Figure 4 confirms the retention of mouse MSCs within the collagen-coated GF after a sustained in vitro culture period. The cells appeared to grow homogenously throughout the entire culture area and appeared to exhibit extensions to connect and network with the substrate (Figure 4(a), depicted by red block arrows). This observation was confirmed by the evidence of prestained cells retained within the collagen-coated GF (Figure 4(b)). The extent of cell proliferation was similar in 3D scaffolds and in control wells when analyzed after 48 hr (4942 ± 1172 cells in 3D scaffolds; 5903 ± 634 cells in 2D wells) and showed no statistically significant differences (*p* = 0.05). These results provide an important basis for the development of the GF as a biocompatible substrate for cell culture, therapy, and tissue engineering.

**Figure 5:**
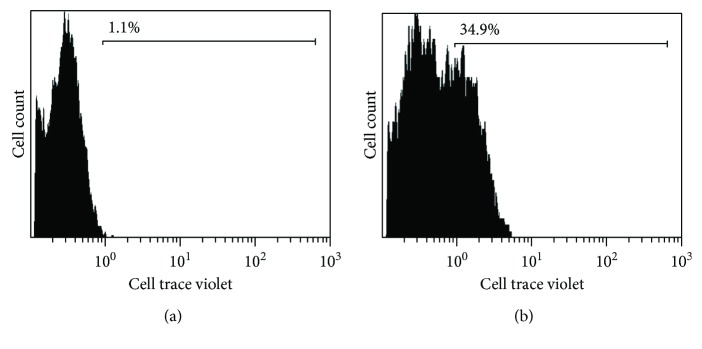
Viability and proliferation of mesenchymal stem cells in collagen-coated GF by FACS analysis. Cells prestained with cell trace violet were cultured up to (a) 24 hr and (b) 48 hr within the collagen-coated GF.

Results from FACS analysis (Figure 5) showed that after 24 hr of culture, 1.1% of the total number of cells seeded had proliferated in comparison to controls (unstained, 0.3%). After 48 hr of culture, 34.9% of the cells were found to have proliferated (in comparison with a 32% proliferating cell population in positive controls). Further, the occurrence of multiple peaks (Figure 5(b)) revealed the presence of consecutive proliferating generations of cells, confirming that the GF was not cytotoxic and promoted mouse MSC adhesion and growth.

For the first time in this study, we adopted and optimized a differentiation protocol for the induction of DA neuronal differentiation of mouse MSCs based on other published protocols using human MSCs (Supplementary Figure 2) [28]. The protocol used a cocktail including sonic hedgehog (SHH), fibroblast growth factor 8 (FGF8), and basic fibroblast growth factor (bFGF) [28]. Further maturation of DA neuronal precursors obtained was promoted by treatment with brain-derived neurotrophic factor (BDNF). This protocol promoted the induction of both human and mouse MSCs to specific transdifferentiated cells, such as DA neurons [28]. We succeeded in reducing the overall differentiation protocol duration from 12 days to 9 days in mouse MSCs, compared with human MSCs. This would allow results to be achieved rapidly compared to previously reported literature [28]. Prior to differentiation, both cell types, human and mouse MSCs, were validated for the maintenance of pluripotency using the ALP assay (Supplementary Figures 3 and 4).

Numerous studies have indicated that various growth factors are involved in the differentiation of embryonic cells into dopaminergic neurons [37]. In addition, SHH and FGF8 when administered simultaneously induce the expression of dopamine-related proteins [38, 39]. Data obtained by Shah et al. in a study where they used GO-based coatings on nanofiber scaffolds to promote oligodendrocyte differentiation from neural stem cells [40] suggested a role for specific microenvironmental interactions that lead to activation of integrin-related intracellular signaling.

**Figure 6:**
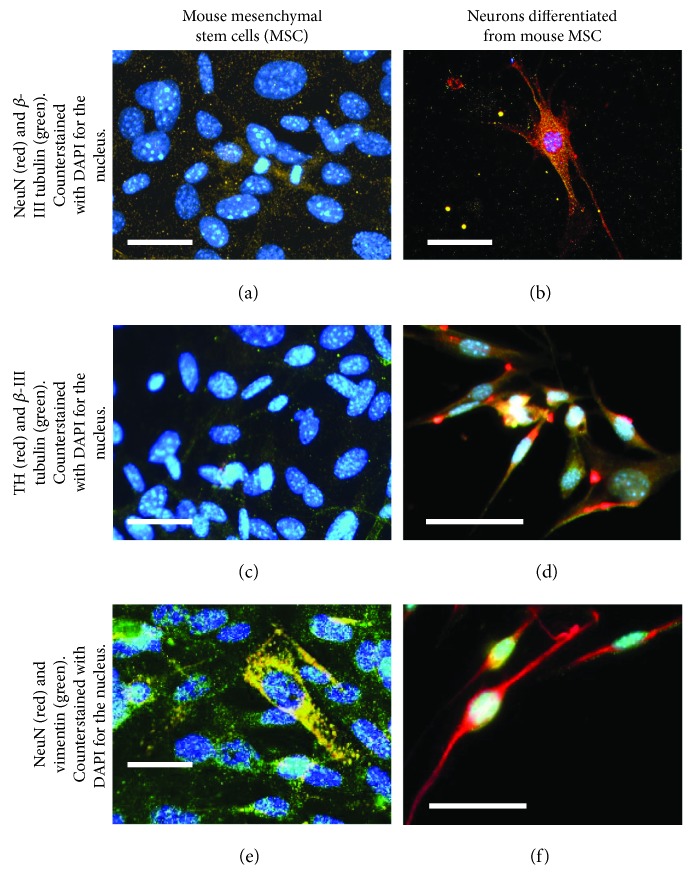
Confirmation of differentiation of mouse mesenchymal stem cells (MSCs) into a neuronal phenotype as they stained positively for (b) *β*-III tubulin and NeuN. These differentiated neurons exhibited a phenotype resembling DA neurons as they positively stained for (d) tyrosine hydroxylase (TH) and *β*-III tubulin. Further, these differentiated DA neurons did not stain positively for (f) vimentin. Controls consisting of undifferentiated mouse MSCs did not stain positively for (a) NeuN and *β*-III tubulin and (c) TH and *β*-III tubulin, but they stained positively for (e) vimentin. Scale bar is 100 *μ*m in all images.

The confirmation of differentiation of mouse MSCs into neurons, stained by the neuronal markers, *β*-III tubulin [41] and NeuN [42], was clearly evident (Figures 6(b) and 6(f)) by atypical neuronal cell-like morphology and extensions, in comparison with controls (Figures 6(a) and 6(e)). It should also be noted that these differentiated neurons expressed a phenotype resembling DA neurons due to their positive expression and enhanced levels of TH [43], unlike their undifferentiated controls (Figures 6(c) and 6(d)). Although the undifferentiated mouse MSCs did not express neuron-like morphology or neuronal markers (Figures 6(a), 6(c), and 6(e)), they stained positively for vimentin, a stem cell marker [44]. These results collectively confirmed the differentiation of mouse MSCs into DA neurons.

**Figure 7:**
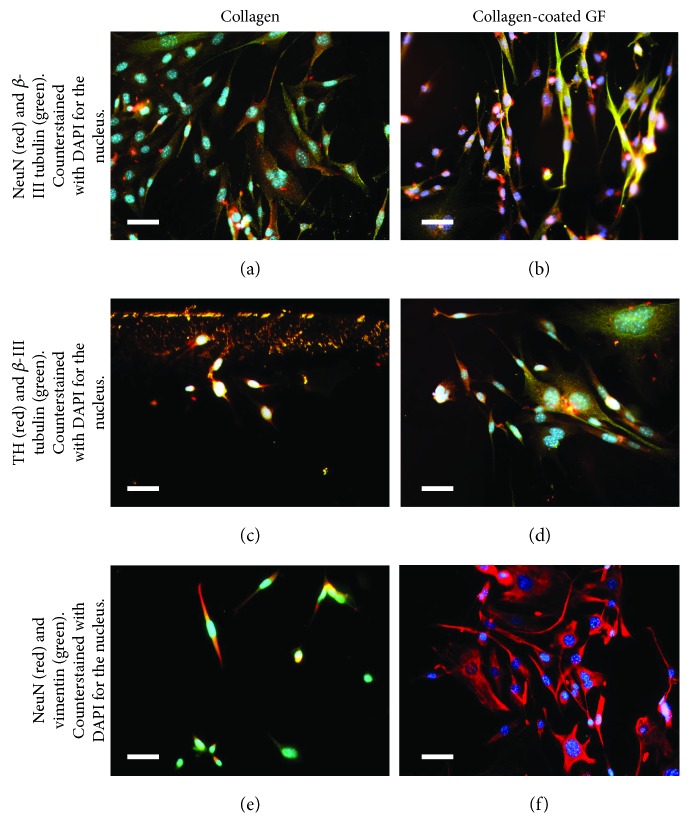
Comparison of differentiated DA neurons with mouse MSCs in contact with (a, c, e) collagen gels and in contact with (b, d, f) collagen-coated GF. Confirmation of differentiation of mouse mesenchymal stem cells (MSCs) into a neuronal phenotype resembling DA neurons was exhibited in all cases, but cells differentiated in graphene foam-based scaffolds exhibited significantly longer neurite extensions than those cultured in contact with collagen. Scale bar is 30 *μ*m in all images.

A comparison of the morphology and expression of neuronal- and dopamine-producing markers in neurons differentiated in both collagen-coated GF and collagen gels revealed significant differences (Figure 7). Cells in contact with the collagen-coated GF showed enhanced expression of not only neuronal markers *β*-III tubulin and NeuN (Figures 7(b), 7(d), and 7(f)) but also TH (Figure 7(d)) in comparison with cells in contact with collagen gels (Figures 7(a), 7(c), and 7(e)).

**Figure 8:**
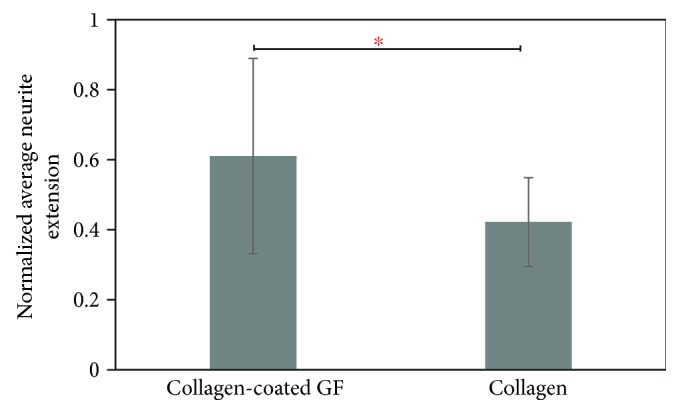
Comparison of normalized average neurite extension length between cells differentiated in collagen-coated GF and collagen only. ^∗^ indicates that the difference between the plotted values was statistically significant.

Comparison of normalized average neurite extension from cells differentiated in both collagen-coated GF and collagen revealed significant differences (*p* = 0.002) (Figure 8). This result implies that the 3D GF might be a better substrate for neuronal culture and differentiation, compared with collagen gels. The images of the neuronal extensions within the 3D scaffolds corresponded to other published images of neuronal networks [45].

Although monolayer graphene substrates have been used for MSC culture [17], it is the GF that offers a 3D porous substrate for neural cell culture and neural regeneration as also shown by others [46]. After coating the GF with collagen, this hybrid scaffold poses as a biocompatible, porous substrate which is effective for cell culture and differentiation. Although several types of polymer-enriched GF have been fabricated [47], this is the first time that GF coated with collagen was prepared and used for neural tissue engineering applications. In the future, specific growth factors could be encapsulated within these scaffolds for delivery to cultured cells for their growth or targeted differentiation [48]. As the 3D GF allows the DA neurons to maintain their morphology and function, we envision that this neuron-filled scaffold can be directly implanted in vivo or be used for studies exploring neuronal functions in vitro in the future.

## 4. Conclusions

Networks of neurons (in vivo) develop via an elaborate succession of cellular events that, when disrupted, can lead to neuron dysfunction and degeneration [3, 8, 10, 18, 49–57]. Injuries or disease conditions, either in the peripheral nervous system (PNS) or central nervous system (CNS), require reconstruction through advanced regenerative medicine and/or tissue engineering approaches. In this study, our goal was to prepare a 3D scaffold suitable for MSC adhesion, growth, and differentiation into DA neurons. We successfully prepared collagen-coated GF as 3D scaffolds for cell culture and differentiation. The collagen coating did not alter the basic properties of the GF but enhanced its hydrophilicity and handling characteristics at the same time. Mouse MSCs adhered and proliferated well within these scaffolds. Furthermore, these MSCs were efficiently differentiated into DA neurons when seeded within these collagen-coated GF.

The outcomes from this study are both novel and significant because it will help reveal interactions between healthy DA neurons and their synaptic communications. It can also help predict mechanisms involved in injury- or disease-induced DA neuron apoptosis. Outcomes from this study can be extended to model other networks of neurons, such as cortical neurons, to study normal and abnormal corticogenesis in the CNS [54], peripheral nerves that are involved in spinal cord injuries [58], or even used to model diabetic neuropathy in vitro [59]. Online supplementary information is provided separately.

## Data Availability

The data used to support the findings of this study are available from the corresponding author upon request.

## Supplementary Materials

Supplementary Materials This section includes Supplementary Figures 1–4 and also contains descriptions of culture and differentiation of human MSCs into DA neurons and the detection of pluripotency markers. References for this section are included in it as well.
