# Supplementary material for: The Efficacy of Graphene Foams for Culturing Mesenchymal Stem Cells and Their Differentiation into Dopaminergic Neurons
Source: Stem Cells Int. 2018 Jun 3;2018:3410168. doi: 10.1155/2018/3410168 (PMC6008666; doi:10.1155/2018/3410168)
Supplement: Supplementary Materials — This section includes Supplementary Figures 1–4 and also contains descriptions of culture and differentiation of human MSCs into DA neurons and the detection of pluripotency markers. References for this section are included in it as well. [file 3410168.f1.zip › Supplementary Figure 3_SCI_2329090.pptx]

## Slide 1
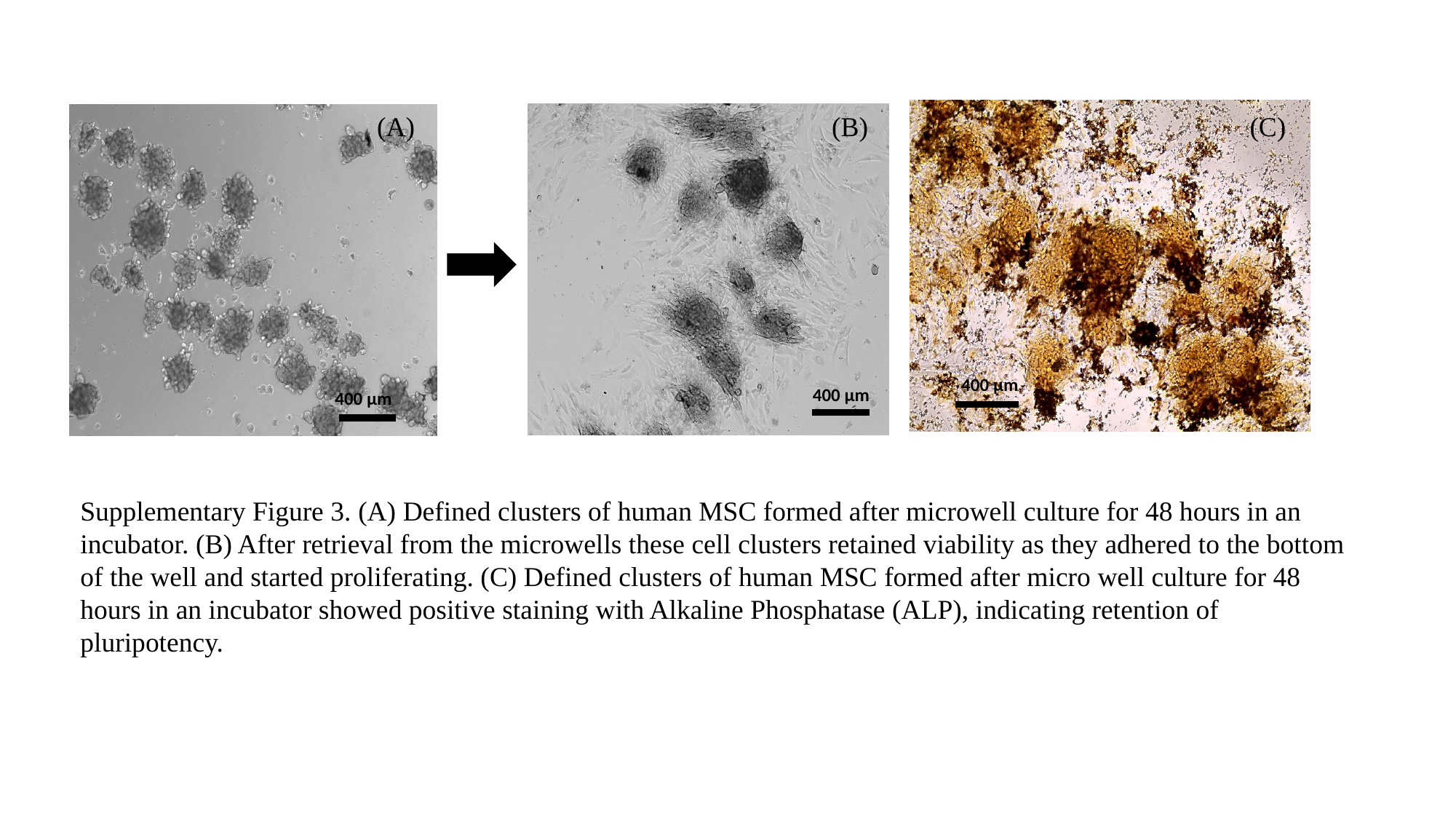

(A)
(B)
(C)
400 µm
400 µm
400 µm
Supplementary Figure 3. (A) Defined clusters of human MSC formed after microwell culture for 48 hours in an incubator. (B) After retrieval from the microwells these cell clusters retained viability as they adhered to the bottom of the well and started proliferating. (C) Defined clusters of human MSC formed after micro well culture for 48 hours in an incubator showed positive staining with Alkaline Phosphatase (ALP), indicating retention of pluripotency.
