# Supplementary material for: The Efficacy of Graphene Foams for Culturing Mesenchymal Stem Cells and Their Differentiation into Dopaminergic Neurons
Source: Stem Cells Int. 2018 Jun 3;2018:3410168. doi: 10.1155/2018/3410168 (PMC6008666; doi:10.1155/2018/3410168)
Supplement: Supplementary Materials — This section includes Supplementary Figures 1–4 and also contains descriptions of culture and differentiation of human MSCs into DA neurons and the detection of pluripotency markers. References for this section are included in it as well. [file 3410168.f1.zip › Supplementary Figure 2_SCI_2329089.pptx]

## Slide 1
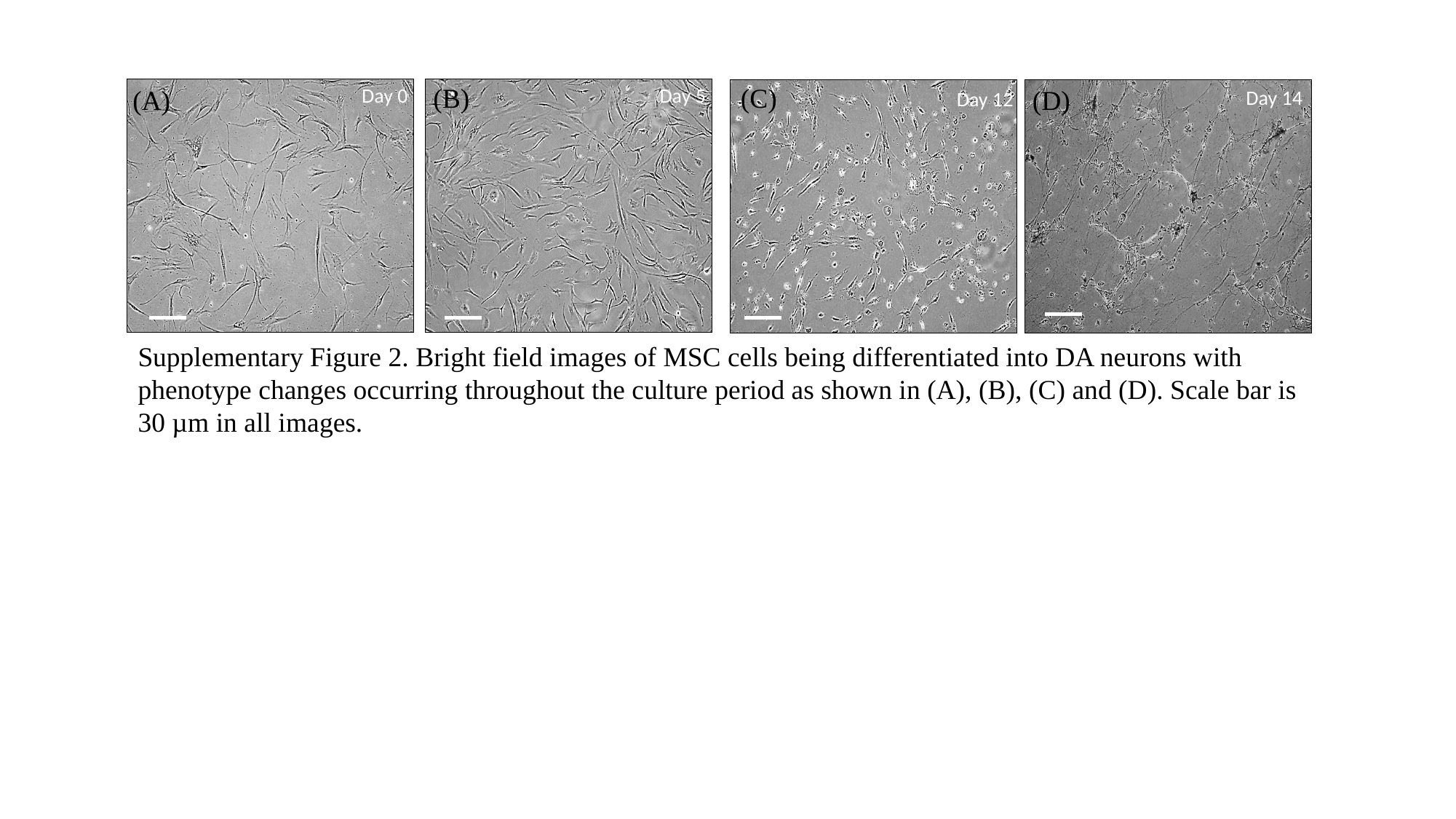

(B)
(C)
Day 0
Day 5
Day 12
Day 14
(A)
(D)
Supplementary Figure 2. Bright field images of MSC cells being differentiated into DA neurons with phenotype changes occurring throughout the culture period as shown in (A), (B), (C) and (D). Scale bar is 30 µm in all images.
