# Supplementary material for: The Efficacy of Graphene Foams for Culturing Mesenchymal Stem Cells and Their Differentiation into Dopaminergic Neurons
Source: Stem Cells Int. 2018 Jun 3;2018:3410168. doi: 10.1155/2018/3410168 (PMC6008666; doi:10.1155/2018/3410168)
Supplement: Supplementary Materials — This section includes Supplementary Figures 1–4 and also contains descriptions of culture and differentiation of human MSCs into DA neurons and the detection of pluripotency markers. References for this section are included in it as well. [file 3410168.f1.zip › Supplementary Figure 1_SCI_2329088.pptx]

## Slide 1
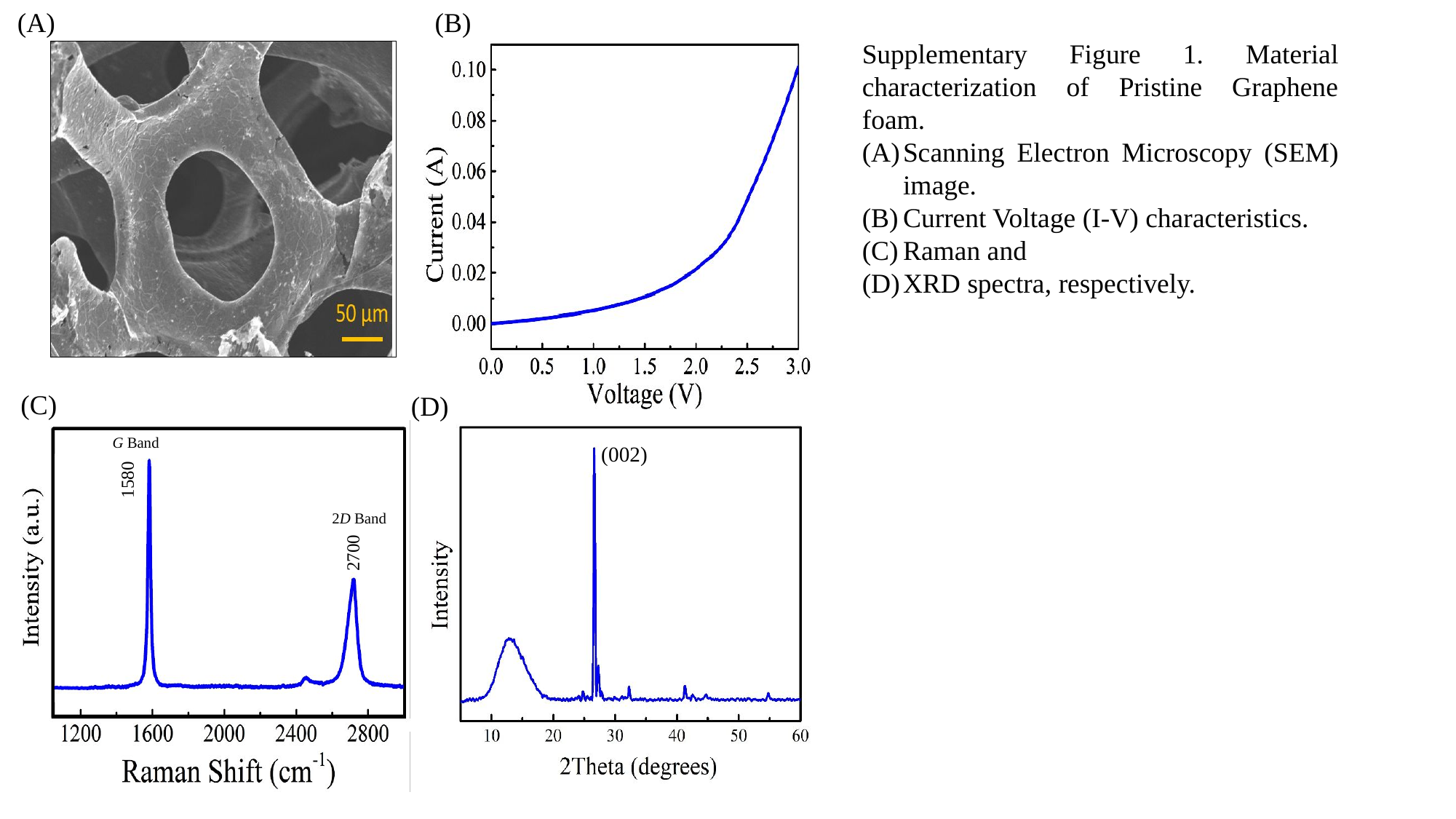

(A)
(B)
Supplementary Figure 1. Material characterization of Pristine Graphene foam.
Scanning Electron Microscopy (SEM) image.
Current Voltage (I-V) characteristics.
Raman and
XRD spectra, respectively.
(C)
(D)
G Band
(002)
1580
2D Band
2700
